# Meta-genetic association of rheumatoid arthritis and *PTPN22 *using PedGenie 2.1

**DOI:** 10.1186/1753-6561-1-s1-s12

**Published:** 2007-12-18

**Authors:** Karen Curtin, Jathine Wong, Kristina Allen-Brady, Nicola J Camp

**Affiliations:** 1 Division of Genetic Epidemiology, Department of Biomedical Informatics, University of Utah School of Medicine, 391 South Chipeta Way, Suite D, Salt Lake City, Utah 84108-1206, USA

## Abstract

PedGenie beta version 2.1 is a unique, flexible, and easily implemented analysis software tool that is enhanced significantly by incorporation of meta-statistics to allow valid combined analysis of multiple studies, including mixtures of family-based and independent resources, in the detection of genetic association with common disease. Genetic Analysis Workshop 15 Problem 2 data, provided by the North American Rheumatoid Arthritis Consortium, were used to demonstrate PedGenie 2.1 meta-association testing of variants in the *PTPN22 *gene and rheumatoid arthritis across multiple resources containing both family-based and independent individuals. Our findings are generally consistent with previous reports for a panel of 14 single-nucleotide polymorphism (SNP) markers, including functional coding SNP R620W, in which the minor allele conferred a significant two-fold increased risk. More power to detect associations was achieved in certain analyses by using extra family-based samples, rather than restricting analyses to single cases randomly selected from each pedigree.

## Background

In the study of common diseases and genes with modest effects, large consortium and multicenter efforts hold the promise of increased power to detect associations, but also present analytical challenges. Candidate gene study populations differ geographically and ethnically, and considerable differences in case-control ascertainment and pedigree structures between resources are likely. Currently, no software package exists that allows valid meta-genetic association testing in mixtures of independent and family-based resources (including pedigrees of arbitrary length and configuration) between or within studies. PedGenie 2.1 (beta version) extends the functionality currently available in PedGenie (version 1.2) [1,2] by incorporating meta-statistics for combined analysis of multistudy resources, along with Monte Carlo significance testing, which allows for a mixture of pedigree members (both nuclear and extended families) and independent individuals. Data from Problem 2 of the Genetic Analysis Workshop 15 (GAW15) were used to demonstrate meta-association testing of the *PTPN22 *candidate gene (14 single-nucleotide polymorphisms, or SNPs) and consensus criteria rheumatoid arthritis (RA) phenotype and sub-phenotypes in combined family-based and independent individuals using PedGenie 2.1. RA, a common systemic autoimmune disease, affects about 1% of adults worldwide and has an estimated heritability of 50 to 60% [3,4]. The association of RA susceptibility with a missense variant in the hematopoietic-specific protein tyrosine phosphatase gene, *PTPN22 *(R620W, rs2476601), has been previously suggested [3].

## Methods

### *PTPN22 *SNP and RA phenotype data

Data provided by the North American Rheumatoid Arthritis Consortium (NARAC) were obtained for 14 SNPs in *PTPN22*. The genotypes and phenotypes were collected from Caucasian individuals in NARAC affected sibling-pair families (1393 cases) and 1519 matched, independent non-diseased controls from New York City (NYC), reported in Carlton et al. [5]. Within this sample were data for 839 affected sib-pair cases and 855 controls, reported in Plenge et al. [6]. In addition to RA status (affected or unaffected), detailed phenotype information available on most cases included rheumatoid factor IgM (RF), a measure of active disease correlated with erosive arthritis. A threshold of 11 and greater was designated as RF+. In Kroot et al. [7] RF titers under 10 were considered normal; however, as levels below 11 could not be quantitated accurately, we used this slightly higher threshold. Elevated anti-cyclic citrullinated peptide (anti-CCP) levels have been shown to predict increased risk for RA development, with an antibody titer threshold of 49 or greater considered anti-CCP+ [4].

### Demonstration studies: NARAC 1 and NARAC 2

For the purpose of demonstrating meta-analysis across multiple studies with PedGenie 2.1 in association testing of a candidate gene, the *PTPN22 *SNP and phenotype data were separated into two study files, designated NARAC 1 and NARAC 2 (Table 1). NARAC 2 comprised the 1694 individuals that were studied by Plenge et al. [6], including both families and independent controls. NARAC 1 comprised all remaining individuals, family cases, unaffected family controls, and independent NYC controls. Because PedGenie can incorporate family relationships in association testing, any individuals were included for whom affected status could be determined and genotypes were available. Specifically, the data analyzed contained siblings and genotyped parents. In two NARAC 1 pedigrees, four adult offspring in affected sibships with genotypes and RA diagnosis between ages 17 to 44 were also available. Including unaffected siblings with genotypes resulted in an additional 103 family-based controls for analysis in NARAC 1.

**Table 1:** *PTPN22 *SNP data: study descriptives

	NARAC Study 1^a^				NARAC Study 2^a^			
		
	Cases		Controls		Cases		Controls	
				
	*N*	%	*N*	%	*N*	%	*N*	%
RA	446		767		839		854	
Family-based	446	100.0	103	13.4	839	100.0	--	--
Independent	--	--	664	86.6	--	--	854	100.0
Age at onset^b^	39	13.9	--	--	39	13.1	--	--
RF+ (≥ 11)	346	77.5	--	--	708	84.4	--	--
RF- (< 11)	89	20.0	--	--	129	15.4	--	--
RF unknown	11	2.5	--	--	2	0.2	--	--
anti-CCP+ (≥ 49)	267	59.9	--	--	545	65.0	--	--
anti-CCP- (< 49)	141	31.6	--	--	274	32.7	--	--
anti-CCP unknown	38	8.5	--	--	20	2.4	--	--

^a^Study 1: 202 pedigrees with genotypes; Study 2: 463 pedigrees with genotypes.

^b^Median

### PedGenie 2.1

Use of family data for allelic or genotype association testing must account for correlations between related individuals to avoid underestimation of variance in a statistic of interest and increased type I error. Several family-based association methods exist, but most are limited by pedigree structure or the statistics that can be performed. Ideally, utilizing all available information on pedigree structure is the most informative approach [1], and, for multi-group efforts, the availability of meta-statistics. PedGenie 2.1, which is a Monte Carlo based method, has been developed with meta-capabilities, which entails the use of study-specific allele or haplotype frequencies and established meta-statistics [8]. Briefly, the Monte Carlo procedure is based on simulating null genotype configurations for each resource and deriving null meta-statistics across resources, achieved as follows. Alleles are estimated within each resource. Alleles are then assigned to founders randomly, in proportion to estimated allele frequencies for the specific resource and a Mendelian gene-drop simulation is performed independent of phenotype; each possible null genotype configuration is used to calculate a null meta-statistic. This is repeated to create an empirical null distribution for significance testing. PedGenie, freely available and easily implemented in a computing environment running Java 1.5 [1], was developed to allow flexibility in hypothesis testing; tests may be constructed for alleles or genotypes in any user-defined grouping, using any reference group as baseline. Several options are available within PedGenie to estimate allele frequencies for the gene-drops. In the NARAC 1 and NARAC 2 resources, there are a large number of relatively small pedigrees and the number of genotyped founders is limited. Therefore, the allele frequencies were estimated from all genotyped individuals. The empirical null distributions were created from 1000 simulations. PedGenie appropriately handles sparse data and missing data structure [1]. By providing information on the number of simulations for which a statistic can be calculated, sparse data is indicated when the number of simulations in which a statistic is calculated is less than the total number of simulations. In the gene drop procedure, individuals missing genotypes for a specific locus are reset to missing, and calculation of test statistics in the simulated data are limited to individuals with observed genotypes [1].

### Meta-statistics

PedGenie beta version 2.1 incorporates meta-statistics to allow valid combined analysis of multiple studies, including family-based resources, in the detection of genetic association with common disease. In epidemiologic studies, data are often collected that can be summarized in three-way contingency tables, the presence or absence of a disease phenotype cross-classified with allele or genotype and a controlling for a third categorical variable (study) which represents combinations of levels of several variables (race, sex, age, etc.) [9]. Meta statistics for genotype, composite genotype, or haplotype analysis across studies currently incorporated in PedGenie 2.1 are based on the generalized Cochran-Mantel-Haenszel (CMH) approach described elsewhere [8,10]. CMH procedures are used to calculate odds ratios, chi-squared general association test of independence, and chi-squared test of trend (mean score statistic where ordered wild-type, heterozygous, and homozygous variant genotypes lie on an ordinal scale).

## Results

The study characteristics and RA phenotypes used to demonstrate PedGenie 2.1 meta-association analysis are described in Table 1. Allele frequencies for controls and *PTPN22 *SNP associations with RA for each demonstration study and the combined study PedGenie 2.1 meta-analysis results are shown in Table 2, along with previously published reports. Generally, our meta-analysis results using GAW15 data corroborate previous findings for the panel of 14 SNP markers that includes functional coding SNP R620W [5,6]. It is of note that on inspection of linkage disequilibrium (LD) between SNPs, R620W was not in strong pairwise LD with any other SNP in the set. Markers rs1217413, rs1217388, rs1310182, and rs1217414 were in pairwise LD (measured by *r*^2 ^> 0.4) with each other. SNPs rs12730735 and rs12760457 were in complete LD. SNP ss38346943, which has a rare, protective minor allele, was not in LD with any other marker.

**Table 2:** Allele-based associations with RA (case-control comparison, odds ratios (ORs) estimated for minor allele)

	PedGenie 2.1													
				
	NARAC Study 1^a^				NARAC Study 2^b^				Meta-analysis			Previously published^c,d^		
				
*PTPN22*	MAF^e^	OR	95% CI	*p*^f^	MAF^e^	OR	95% CI	*p*^f^	OR	95% CI	*p*^g^	OR	95% CI	*p*^h^
Rs3789604	0.182	1.11	0.88–1.41	0.36	0.175	1.15	0.95–1.39	0.17	1.13	0.98–1.32	0.10	1.15	0.97–1.35	0.11
Rs3811021	0.182	1.11	0.88–1.40	0.38	0.176	1.14	0.95–1.37	0.17	1.13	0.97–1.31	0.11	1.15	0.97–1.36	0.10
rs1217413	0.210	1.38	1.13–1.70	**0.003**	0.230	1.28	1.07–1.53	**0.002**	1.32	1.16–1.51	**<0.001**	1.37	1.18–1.60	**<0.001**
ss38346942	0.015	1.21	0.61–2.37	0.59	0.012	1.22	0.65–2.27	0.56	1.21	0.75–1.97	0.42	1.03	0.59–1.79	1.00
rs1217388	0.246	1.29	1.04–1.59	**0.017**	0.260	1.20	1.01–1.42	**0.028**	1.23	1.07–1.42	**0.003**	1.28	1.11–1.49	**0.001**
ss38346943	0.031	0.32	0.16–0.67	**0.001**	0.023	0.84	0.47–1.48	0.55	0.60	0.39–0.92	**0.020**	0.63	0.40–1.01	0.05
rs1310182	0.430	1.28	1.06–1.56	**0.008**	0.435	1.28	1.09–1.49	**0.001**	1.28	1.13–1.45	**<0.001**	1.35	1.18–1.54	**<0.001**
ss38346944	0.029	1.14	0.65–1.99	0.63	0.019	1.37	0.84–2.22	0.24	1.26	0.85–1.85	0.24	1.29	0.87–1.93	0.21

rs2476601	0.087	1.79	1.39–2.30	**<0.001**	0.085	2.19	1.75–2.74	**<0.001**	2.03	1.70–2.42	**<0.001**	1.93	1.58–2.37	**<0.001**
(R620W)												2.184	1.76–2.70	**<0.001**

rs12730735	0.292	0.83	0.68–1.01	0.07	0.295	0.89	0.76–1.04	0.14	0.86	0.76–0.98	**0.025**	0.81	0.70–0.94	**0.006**
rs11102685	0.076	1.21	0.87–1.77	0.26	0.078	1.20	0.93–1.55	0.19	1.20	0.98–1.49	0.09	1.27	1.01–1.60	**0.040**
rs12760457	0.291	0.83	0.68–1.02	0.09	0.296	0.88	0.74–1.05	0.14	0.86	0.76–0.98	**0.022**	0.82	0.71–0.96	**0.010**
rs2488458	0.246	1.29	1.05–1.58	**0.014**	0.260	1.20	1.01–1.41	**0.037**	1.23	1.08–1.41	**0.003**	1.27	1.10–1.48	**0.002**
rs1217414	0.282	0.88	0.70–1.10	0.20	0.270	0.84	0.72–0.99	0.06	0.86	0.76–0.97	**0.018**	0.84	0.72–0.98	**0.020**

^a^Problem 2, 446 family cases and 103 family controls in 202 NARAC pedigrees, 664 independent NYC controls; excludes Plenge et al. [6] samples.

^b^Problem 2, samples in Plenge et al. [6] candidate gene file, 839 family cases in 463 pedigrees and 854 independent NYC controls.

^c^Carlton et al. [5] sample set 2 (661 randomly selected independent cases from NARAC pedigrees and 1322 independent NYC controls).

^d^Plenge et al. [6] second collection; 840 family cases (NARAC) and 867 independent NYC controls: R620W SNP only.

^e^Minor allele frequency in controls.

^f^Empirical confidence intervals and chi-square *p*-values, 1000 simulations; *p *< 0.05 in bold.

^g^Empirical confidence intervals and CMH chi-square general association test, 1000 simulations; *p *< 0.05 in bold.

^h^Fisher's exact chi-square test; *p *< 0.05 in bold.

Table 3 shows genotype associations with RA and *PTPN22 *markers for an additive model (Armitage test for trend [11]) that were significant in at least one study. Previously, Carlton et al. reported two markers with associations independent of R620W; rs3789604 and rs1310182, both in putative transcription factor binding sites [5]. The odds ratios reported by Carlton et al. were adjusted accordingly, and these are shown in Table 3. R620W was adjusted for rs3789604, and rs3789604 and rs1310182 were adjusted for R620W.

**Table 3:** Genotype-based associations with RA (case-control comparison, reference is major allele homozygote)

	PedGenie 2.1												
				
	NARAC Study 1^a^				NARAC Study 2^b^			Meta-analysis			Previously published^c^		
				
*PTPN22*	Genotype^d^	OR	95% CI	*p*-trend^e^	OR	95% CI	*p*-trend^e^	OR	95% CI	*p*-trend^f^	OR	95% CI	*p*-trend
rs3789604	AC	0.94	0.69–1.26		1.16	0.93–1.46		1.07	0.91–1.26		1.15	0.93–1.42	
	CC	1.68	0.83–3.38	0.39	1.25	0.70–2.21	0.18	1.43	0.93–2.18	0.12	1.78	1.13–2.79	**0.014**
rs1217413	TC	1.44	1.11–1.87		1.34	1.08–1.66		1.38	1.17–1.63				
	CC	1.71	1.06–2.75	**0.003**	1.52	1.00–2.31	**0.002**	1.59	1.14–2.22	**<0.001**			
rs1217388	TC	1.49	1.14–1.93		1.30	1.05–1.61		1.37	1.15–1.63				
	CC	1.30	0.78–2.17	**0.017**	1.27	0.86–1.87	**0.026**	1.28	0.94–1.74	**0.003**			
ss38346943	TC	0.32	0.15–0.69		0.88	0.48–1.61		0.61	0.39–0.95				
	CC	--	--	--	--	--	--	--	--	--			
rs1310182	CT	1.55	1.13–2.13		1.39	1.11–1.73		1.45	1.20–1.75		1.22	0.98–1.77	
	TT	1.57	1.07–2.30	**0.011**	1.62	1.18–2.22	**<0.01**	1.60	1.25–2.03	**<0.001**	1.32	0.97–1.54	0.052

rs2476601	CT	1.92	1.41–2.60		2.36	1.84–3.04		2.18	1.80–2.65		2.19	1.73–2.78	
(R620W)	TT	1.96	0.74–5.21	**<0.001**	2.91	1.26–6.74	**<0.001**	2.53	1.32–4.84	**<0.001**	2.90	1.37–6.18	**<0.001**

rs12730735	AG	1.00	0.75–1.31		1.10	0.89–1.36		1.06	0.89–1.25				
	GG	0.48	0.29–0.80	0.08	0.56	0.37–0.83	0.14	0.53	0.39–0.72	**0.024**			
rs12760457	AG	1.01	0.75–1.36		1.09	0.87–1.37		1.06	0.89–1.26				
	GG	0.48	0.28–0.82	0.09	0.55	0.37–0.82	0.14	0.52	0.38–0.72	**0.023**			
rs2488458	GA	1.51	1.15–1.97		1.33	1.07–1.66		1.40	1.18–1.67				
	AA	1.27	0.79–2.05	**0.014**	1.20	0.81–1.78	**0.037**	1.23	0.91–1.67	**0.003**			
rs1217414	CT	1.02	0.77–1.35		0.85	0.69–1.04		0.91	0.78–1.06				
	TT	0.60	0.34–1.03	0.20	0.70	0.46–1.06	0.053	0.65	0.46–0.92	**0.018**			

^a^Problem 2 data, 446 family cases and 103 family controls in 202 affected NARAC pedigrees, 664 independent NYC controls; excludes Plenge et al. [6] samples.

^b^Problem 2 data, samples in Plenge et al. [6] candidate gene file, 839 family cases in 463 pedigrees and 854 independent NYC controls.

^c^Carlton et al. [5] sample set 2, 661 random independent cases from NARAC pedigrees and 1322 NYC controls; adjusted for R620W or rs3789604 (R620W).

^d^Minor allele heterozygote and homozygote genotypes according to Carlton et al. [5] Table 1.

^e^Empirical confidence intervals and chi-square *p*-values, 1000 simulations; *p *< 0.05 in bold.

^f^Empirical confidence intervals and CMH chi-square trend test *p*-values, 1000 simulations; *p *< 0.05 in bold.

Previous studies reported that susceptible *PTPN22 *R620W genotypes containing the variant allele were strongly associated with RF+ but not RF- disease [3,6]. Our meta-analysis supported an association in RF+ cases only compared to controls, although a comparison of RF+ to RF- cases was not significant (Table 4). Plenge et al. reported an association with R620W variant genotypes and anti-CCP+ cases, but not in anti-CCP- cases [6]. Our meta-analysis showed an association for anti-CCP+ cases and anti-CCP- cases in comparison to controls (Table 4). Thus, in our larger meta-analysis across studies, we could not confirm that anti-CCP level discriminates in R620W-associated RA. However, an association was seen for SNPs in pairwise LD (rs1217413, rs1217388, rs1310182, and rs2488458) and anti-CCP+ cases only vs. controls (heterozygous/variant vs. wild-type odds ratios (ORs) between 1.4 and 1.5, 95% confidence interval (CI), 1.2 to 1.8). Further, the comparison of anti-CCP+ to anti-CCP- cases was significant for these markers.

**Table 4:** Genotype-based associations with R620W and RA sub-type (reference is major allele CC homozygote)

		PedGenie 2.1											
					
		NARAC Study 1^a^			NARAC Study 2^b^			Meta-analysis			Previously published^c^		
					
RA Phenotype	Genotype	OR	95% CI	*p*^d^	OR	95% CI	*p*^d^	OR	95% CI	*p*^e^	OR	95% CI	*p*
RF+ vs. Ctrls.	CT or TT	1.80	1.29–2.49	**<0.001**	2.34	1.82–3.00	**<0.001**	2.13	1.76–2.57	**<0.001**	2.36	1.76–3.16	**<0.001**
RF- vs. Ctrls.	CT or TT	1.40	0.87–2.27	0.20	1.08	0.71–1.65	0.77	1.20	0.87–1.65	0.31	1.17	0.6–2.31	0.64
RF+ vs. RF-	CT or TT	1.07	0.58–2.00	0.79	1.46	0.83–2.56	0.12	1.30	0.86–1.95	0.18			
											
											Previously published^f^		
											
CCP+ vs. Ctrls.	CT or TT	1.69	1.18–2.41	**0.001**	2.03	1.57–2.62	**<0.001**	1.90	1.55–2.34	**<0.001**	1.49	1.23–1.79	**<0.001**
CCP- vs. Ctrls.	CT or TT	1.59	1.04–2.45	**0.035**	1.41	1.03–1.95	**0.029**	1.47	1.15–1.88	**0.001**	1.04	0.83–1.29	0.40
CCP+ vs. CCP-	CT or TT	1.05	0.61–1.80	0.93	1.16	0.83–1.60	0.32	1.12	0.84–1.50	0.47			
RF or CCP+ vs. RF-/CCP-	CT or TT	1.29	0.65–2.53	0.55	1.43	0.81–2.52	0.06	1.38	0.89–2.13	0.051	1.43	1.16–1.77	**<0.001**

^a^Problem 2 data, 446 family cases and 103 family controls in 202 affected NARAC pedigrees, 664 independent NYC controls; excludes Plenge et al. [6] samples.

^b^Problem 2 data, samples in Plenge et al. [6] candidate gene file, 839 family cases in 463 pedigrees and 854 independent NYC controls.

^c^Begovich et al. [3] replication sample (NARAC; 463 independent-sib cases, 926 independent controls), CLR analysis.

^d^Empirical confidence intervals and chi-square *p*-values, 1000 simulations; *p *< 0.05 in bold.

^e^Empirical confidence intervals and CMH chi-square general association test, 1000 simulations; *p *< 0.05 in bold.

^f^Plenge et al. [6] first clinical collection, Epidemiological Investigation of RA (EIRA) Swedish cohort (2370 cases 1757 controls); allele association.

## Discussion

PedGenie 2.1 can correct for all relationships in family-based resources, therefore all family members with phenotype and genotype data are available can be included. If only one affected sibling was chosen from each pedigree, the sample size here would have reduced from 1285 cases and 1621 controls to 665 and 1518, respectively. For example, in NARAC 1, a family with five affected and five unaffected genotyped siblings and parents with known RA status (one genotyped) were included in PedGenie analyses, whereas only one affected sib was randomly selected from this family for the study performed by Carlton et al. [5].

CMH statistics for ordered genotypes have been used to assess association in multiple case-control studies in which cases are independent, either probands or randomly selected affecteds [12]. A combined odds ratio estimate of the association in both case-control and transmission-disequilibrium studies have been proposed [13]. PedGenie beta 2.1 allows for valid meta-analyses of combined family-based and case-control studies using CMH techniques, while accommodating comprehensive information in large, multigenerational families without pedigree splitting required in other packages. The ability to combine family and case-control resources and use all data available both increases the utility of prior linkage resources and can provide increased power to detect associations, particularly in stratified and subset analyses that likely lead to small sample sizes in individual studies.

In conclusion, our method is a more comprehensive way of using all data available in meta-association testing, with more power to detect associations by using extra family-based samples rather than restricting to randomly selected cases from each pedigree. Our findings generally corroborate those previously reported. We support previous findings that the *PTPN22 *gene is associated with RA. However, our results do not indicate that anti-CCP antibody status significantly discriminates for disease in the functional R620W SNP.

## Competing interests

The author(s) declare that they have no competing interests.
